# Early Onset Diabetes in Two Children due to Progeria, a Monogenic Disease of DNA Repair

**DOI:** 10.4274/jcrpe.galenos.2019.2019.0126

**Published:** 2020-09-02

**Authors:** Martin Holder, Valerie Schwitzgebel

**Affiliations:** 1 Klinikum Stuttgart, Olgahospital, Department of Pediatric Endocrinology and Diabetology, Stuttgart, Germany; 2 Hopital des Enfants, Endocrinologie et Diabetologie Pediatriques, Geneve, Switzerland

**Keywords:** Progeria syndrome, diabetes mellitus, metformin, prevention

## Abstract

Progeria syndrome is a rare disorder in childhood which causes accelerated systemic aging. Due to the accelerated aging process, disorders which normally occur only in old age will appear in these children at a much younger age. We report two children with progeria syndrome, in whom fulminant diabetes mellitus manifested at a very early age.

**What is already known on this topic?**

Less is known about type 2 like diabetes mellitus in children and adolescents with progeria-syndrome although they have a high risk of developing diabetes mellitus.

**What this study adds?**

Early and regular screening for diabetes mellitus are mandatory. Treatment with metformin at an early stage should be recommended to prevent early symptoms of diabetes and potentially delay the clinical course of progeria.

## Introduction

Progeria syndrome is a group of very rare genetic disorders which are characterized by premature aging and classified by various names based on causative etiology: Hutchinson-Gilford progeria syndrome (HGPS), Néstor-Guillermo progeria syndrome, atypical progeria syndromes, restrictive dermopathy, mandibuloacral dysplasia, Werner syndrome (WS), Bloom syndrome, Rothmund-Thomson syndrome, Cockayne syndrome (CS), xeroderma pigmentosum, trichothiodystrophy, Fanconi anaemia, Seckel syndrome, ataxia telangiectasia (AT), AT-like disorder, cerebroretinal microangiopathy with calcifications and cysts, and Nijmegen breakage syndrome.

Children affected by progeria syndrome appear normal at birth, but the clinical manifestations become apparent in the first few years of life. Manifestations of progeria syndrome include failure to thrive, dermatologic, musculoskeletal, and neurologic abnormalities and eventually life-limiting cardiovascular disease can occur. Additionally, they may have audiologic, dental, and ophthalmologic issues that impair their lives. Less is known about metabolic complications in children with progeria syndrome. In WS, also known as adult progeroid syndrome, type 2 like diabetes mellitus is one of the clinical manifestations of the disease and attention must give to the differential diagnosis (1).

We report two patients, a boy and a girl, with progeria syndrome in whom fulminant diabetes mellitus manifested very early.

## Case Reports

### Case 1

### Boy with Cockayne Syndrome

The boy was born per section on the 38^th^ week of pregnancy with a birth weight of 2,250 g (1^st^ percentile, z-score -2.42), 41 cm length (<1^st^ percentile, z-score -4.39) and 31 cm head circumference (<1^st^ percentile, z-score -2.79). Within the first year delayed motor development, particularly of the gross motor skills, and delayed linguistic development became evident.

At the age of 22 months the boy was first seen in our social pediatric department due to severe psychomotor retardation, microcephaly and high-grade dystrophic macrosomia. In the following years CS was diagnosed in an external department. CS is one of the progeroid syndromes. At the age of seven years he was severely handicapped with hepatopathy, leukodystrophy and a spastic tetraparesis affecting predominantly the lower limbs (see Table 1). Nutrition was administered via a percutaneous endoscopic gastrostomy. Five days before presenting with diabetes in our department his antiepileptic medication was changed to Levetiracetam. During the previous day he had become progressively weaker and was unusually restless throughout the night with abnormal arm movements.

He presented acutely at the age of seven years with severe hyperglycemia due to hyperglycemic - hyperosmolar syndrome (HHS). This syndrome is characterized by extreme elevations in serum glucose concentrations, hyperosmolality without significant ketosis and a high mortality (2).

The criteria for HHS include: Plasma glucose concentration >600 mg/dL (33.3 mmol/L), venous pH *>*7.25, arterial pH *>*7.30, serum bicarbonate *>*15 mmol/L, slight ketonuria, absent to mild ketonemia, effective serum osmolality *>*320 mosmol/kg and altered consciousness (e.g., obtunded, aggressive) or seizures.

At that time his weight was 8700 g (approx. 12 kg below the third percentile), his length 86 cm (approx. 30 cm below the third percentile) and hypertensive RR-values with 174/147 mmHg. The initial plasma glucose level was 925 mg/dL, pH 7.4, HbA1c 7.3% (56.28 mmol/mol), C-peptide 9 ng/mL and serum osmolality 345 mosmol/kg (275-305 mosmol/kg) (see Table 1). As a result of high insulin sensitivity at that stage and to avoid rapid drop in blood sugar with standardized insulin treatment and development of hypernatremia, the patient was managed on the intensive care unit with meticulous rehydration and a gradual, slow reduction in plasma glucose. There insulin was administered intravenously with great care at 0.025-0.05 IE/body weight/hour. After emergency treatment and clinical recovery, the blood glucose levels could be adequately controlled in relation to tube feeding using a rapid acting human insulin three times daily to the feedings and long acting insulin analogues, which could be stopped subsequently. His blood sugar target under treatment was 150-200 mg/dL (8.3-11.1 mmol/L). Under antihypertensive treatment with captopril his RR-values were stable.

### Case 2

### Girl with Hutchinson-Gilford Progeria Syndrome

The girl had an existing diagnosis of HGPS when she first presented to our department at the age of 14 years with severe coronary heart disease and hyporegenerative anemia requiring regular blood transfusions. The diagnosis of HGPS has not been genetically proven as the parents refused genetic tests. At first presentation her weight was 13.6 kg (26 kg below the third percentile), her length was 120 cm (31 cm below the third percentile) and her RR-value was 95/55 mmHg. She showed tachycardia with a pulse rate of 120 per minute and oxygen saturation of 92%. As part of the initial investigations she was found to have elevated blood glucose levels; plasma glucose 324 mg/dL (18 mmol/L), HbA1c 8.6% (70.49 mmol/mol), C-peptide 20.24 ng/mL and insulin 500 mU/L (see Table 1). As the patient had already been receiving palliative care, a decision was made in conjunction with the parents that no further active treatment, neither insulin nor metformin, would be administered.

Both children have subsequently died. Their mortality was not related to diabetes.

## Discussion

The two children described above had progeria syndrome due to different etiologies. Clinically the girl had HPGS and the boy CS. Both children developed partly fulminant type 2-like diabetes mellitus, which is yet not known in these young patients. Only in WS, the adult form of progeria syndrome, diabetes mellitus due to severe insulin resistance has been reported as a possible clinical manifestation. The possible mechanism of insulin resistance includes reduced insulin receptors in fat cells, loss of signal transduction after the binding of normal insulin to normal receptors and a defective post-receptor step (1). In addition, dysregulation of adipocytokine may be another mechanism for the development of diabetes mellitus in WS patients.

HPGS is due to a mutation in the lamin A* (LMNA)* gene that leads to the production of a truncated and toxic form of LMNA, called progerin (3). Progerin accumulates and triggers growth impairment, lipodystrophy, dermal and bone abnormalities and cardiovascular changes, leading to a shortened lifespan. There is a major rationale for targeting progerin at different levels. Attempts to develop treatment in HGPS associated with progerin accumulation may thus rely on a multi-targetted approach, including decreased progerin production, increased degradation, or downstream noxious cascades (4).

In 2011, the RNA-binding protein SRSF1 (serine/arginine-rich splicing factor 1) was shown to affect alternative splicing of *LMNA* in human HGPS primary fibroblasts and mouse LMNA fibroblast (5). A recent whole-genome transcription analysis has revealed that SRSF1 expression is regulated by the anti-diabetic drug metformin (6). In a current study it has been demonstrated that metformin reduced progerin expression by regulating SRSF1 expression and altering the pathological phenotypes of HGPS cells. After treatment with 5 mmol/L of metformin, a decrease in SRSF1 protein of up to 40% could be demonstrated (3). Therefore, it may be interesting to explore the therapeutic potential of metformin in patients with this form of progeria.

Laminopathies, due to mutations in *LMNA*, encoding A type-lamins, can lead to premature ageing but also to lipodystophic syndromes, showing that these diseases may have related physiopathological mechanisms (7). Lipodystrophy syndromes are frequently associated with hormonal and metabolic derangements resulting in severe comorbidities that depend on the subtype, extent of fat loss, age and gender. Many complications of lipodystrophy are secondary to deficient adipose mass, resulting in ectopic lipid storage in the liver, muscle, and other organs and causing severe insulin resistance. Insulin resistance leads to diabetes, hypertriglyceridemia, polycystic ovarian syndrome and non-alcoholic fatty liver disease (8).

The male patient had proven CS resulting in a severe progeria syndrome. Very recently inherited defects in DNA repair have been identified as the underlying cause (9). DNA maintenance is emerging as a central factor in a multitude of diseases and loss of genomic integrity leads to severe multisystem syndromes. Loss of transcription-coupled repair, which occurs with mutations in twofold excision repair of cross-complementing genes (*ERCC6* and *ERCC8*) results in CS, which is characterized by progressive cachexia, severe growth retardation and leukoencephalopathy. Gradually our understanding of the clinical spectrum of the progeroid syndromes is becoming clearer. Clinical trials of treatment for these monogenic DNA-repair disorders may well be the key to intervention in other diseases associated with genomic damage and perhaps even for aging itself (9).

Due to the therapeutic potential of metformin, children and adolescents with progeria syndrome should be screened for diabetes from a very early age onwards and treated with metformin. Metformin is a well-known anti-diabetic drug, which has demonstrated a good safety profile in millions of patients over the past two decades. In children and adolescents with type 2 diabetes, metformin is the recommended first line therapy and is superior to treatment with sulfonylureas (10,11). Metformin acts through adenosine monophosphate (AMP) kinase in liver, muscle, and fat tissue, with a predominant action on the liver. Hepatic glucose output is reduced by decreased gluconeogenesis. Insulin-stimulated glucose uptake is increased in muscle and fat. Long-term use is associated with a 1-2% reduction in HbA1c (11).

Recently, new pathways in addition to AMPK activation – as discussed above – were discovered, which would explain the additional positive properties of metformin (12). The potential use of metformin as an anti-aging drug and its effect on progerin expression may be interesting to explore in the future.

Unfortunately, neither child was treated with metformin. In the case of the male patient, the evidence had not been published concerning the potential effects of metformin on both the metabolic and disease-progressive course. Treatment with metformin was refused by the female patient and her family because of the palliative care status.

However, for other children with progeria syndrome who are at risk of developing early type 2-like diabetes, treatment with metformin at an early stage should be recommended. As a result early symptoms of diabetes may be prevented and the clinical course of progeria potentially delayed.

## Conclusion

Less is known about type 2-like diabetes mellitus in children and adolescents with progeria-syndrome, although they have a high risk of developing diabetes mellitus. Therefore, early and regular screening for diabetes mellitus are mandatory in these patients. Treatment with metformin at an early stage should be recommended which may prevent early symptoms of diabetes and potentially delay the clinical course of progeria.

## Figures and Tables

**Table 1:**
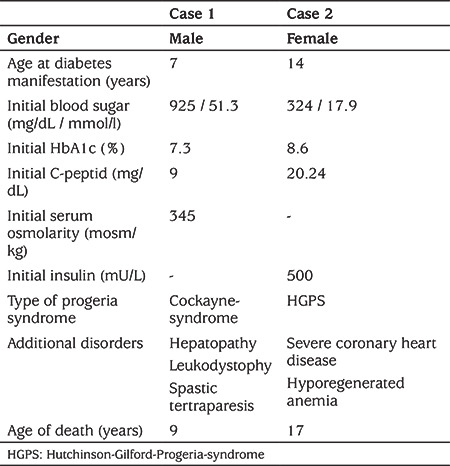
Main characteristics of both patients with Progeria syndrome
